# Discrete distribution of implanted and annealed arsenic atoms in silicon nanowires and its effect on device performance

**DOI:** 10.1186/1556-276X-7-685

**Published:** 2012-12-21

**Authors:** Masashi Uematsu, Kohei M Itoh, Gennady Mil'nikov, Hideki Minari, Nobuya Mori

**Affiliations:** 1 School of Fundamental Science and Technology, Keio University, Yokohama, 223-8522, Japan; 2 Graduate School of Engineering, Osaka University, Osaka, 565-0871, Japan; 3 CREST, JST, Tokyo, 102-0075, Japan

**Keywords:** Silicon nanowires, Random discrete dopant distribution, Gate-all-around transistors, Kinetic Monte Carlo, Non-equilibrium green's function

## Abstract

We have theoretically investigated the effects of random discrete distribution of implanted and annealed arsenic (As) atoms on device characteristics of silicon nanowire (Si NW) transistors. Kinetic Monte Carlo simulation is used for generating realistic random distribution of active As atoms in Si NWs. The active As distributions obtained through the kinetic Monte Carlo simulation are introduced into the source and drain extensions of n-type gate-all-around NW transistors. The current–voltage characteristics are calculated using the non-equilibrium Green's function method. The calculated results show significant fluctuation of the drain current. We examine the correlation between the drain current fluctuation and the factors related to random As distributions. We found that the fluctuation of the number of dopants in the source and drain extensions has little effect on the on-current fluctuation. We also found that the on-current fluctuation mainly originated from the randomness of interatomic distances of As atoms and hence is inherent in ultra-small NW transistors.

## Background

Fluctuation due to random discrete dopant (RDD) distribution is becoming a major concern for continuously scaled down metal-oxide semiconductor field-effect transistors (MOSFETs) [1-4]. For ultra-small MOSFETs, not only random location of individual dopant atoms but also fluctuation of the number of active impurities is expected to have significant impacts on the device performance. Effects of the RDD distribution are usually analyzed with a randomly generated RDD distribution. The actual RDD distribution, however, should be correlated with the process condition and can be different from a mathematically generated one. In the present study, we investigate the effects of random discrete distribution of implanted and annealed arsenic (As) atoms in source and drain (S/D) extensions on the characteristics of n-type gate-all-around (GAA) silicon nanowire (Si NW) transistors. We investigate a GAA Si NW transistor since it is considered as a promising structure for ultimately scaled CMOS because of its excellent gate control [2,5-7]. Kinetic Monte Carlo (KMC) simulation is used for generating realistic random distribution of active As atoms in Si NWs. The current–voltage characteristics are then calculated using the non-equilibrium Green's function (NEGF) method. Our results demonstrate that the on-current fluctuation mainly originated from the randomness of the dopant location and hence is inherent in ultra-small NW transistors.

## Methods

**Figure 1:**
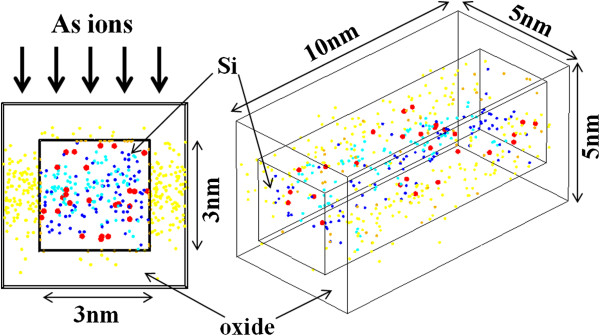
**Discrete As distribution in a Si NW.** Cross-sectional view (left) and entire view (right). Red dots show active As atoms in Si. Blue dots, As precipitates; light blue dots, As-V clusters; orange dots, As at the oxide/Si interface; and yellow dots, As in the oxide.

**Figure 2:**
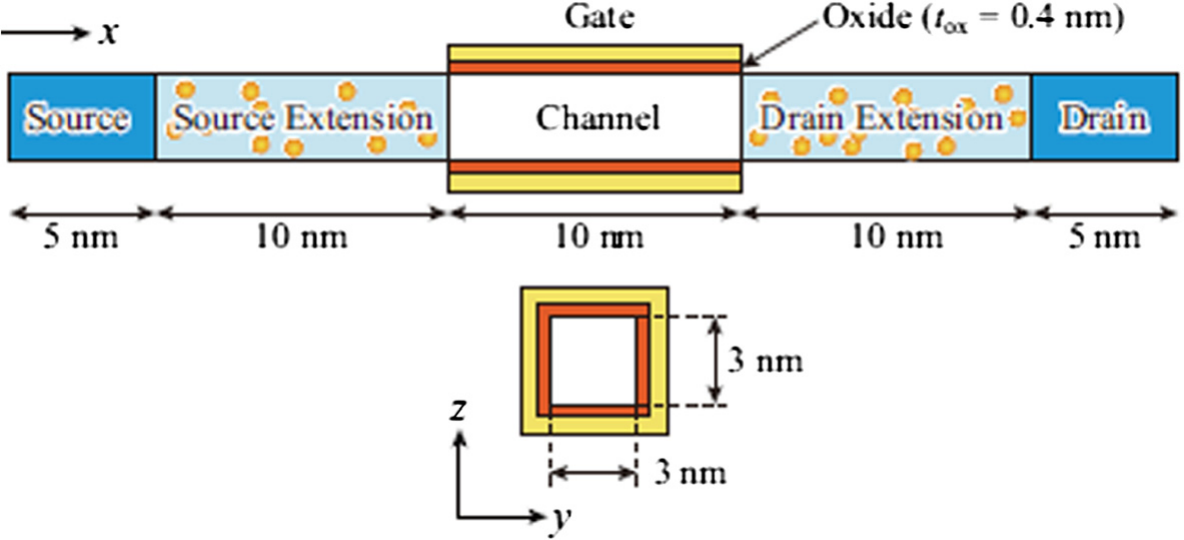
**Schematic diagram of the n-type GAA Si NW MOSFET.** Discrete distributions of the active As atoms are introduced into the S/D extensions. To mimic metal electrodes, the S/D regions are heavily doped with *N*_d_ = 5 × 10^20^ cm^−3^ (continuously doping). The channel region is intrinsic. We simulated 100 samples using 200 different random seeds (each sample needs two random seeds for S/D extensions).

Random discrete As distribution in a Si NW is calculated using Sentaurus KMC simulator (Synopsys, Inc., Mountain View, CA, USA) [8-10]. Figure 1 shows an example of the calculated discrete As distribution in a Si NW (3 nm wide, 3 nm high, and 10 nm long) with 1-nm-thick oxide. The Si NW is implanted with As (0.5 keV, 1 × 10^15^ cm^−2^) and annealed at 1,000°C with a hold time of 0 s. Statistical variations are investigated using 200 different random seeds. The active As distributions obtained through the KMC simulation are then introduced into the S/D extensions of n-type Si NW MOSFETs, whose device structure is given in Figure 2. In the present study, we consider only an intrinsic channel, and impacts of possible penetration of dopant atoms into the channel region are not examined. To mimic metal electrodes, the S/D regions are heavily doped with *N*_d_ = 5 × 10^20^ cm^−3^ (continuously doping). We simulate 100 samples using 200 different random seeds (each sample needs two random seeds for S/D extensions). The drain current-gate voltage (*I*_d_*V*_g_) characteristics are calculated using the NEGF method with an effective mass approximation [11,12]. The discrete impurities are treated with a cloud-in-cell charge assignment scheme [13]. Phonon scattering is not taken into account in the present calculation.

## Results and discussion

### As distribution by KMC simulation

**Figure 3:**
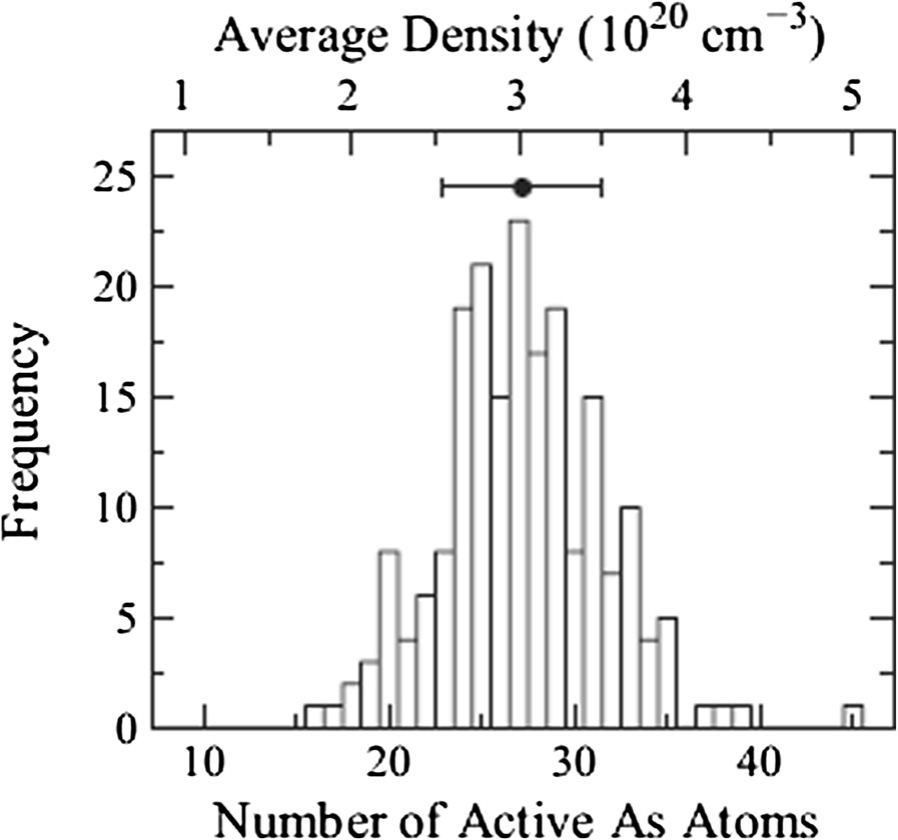
**Histogram of the number of active As atoms in the Si NWs.** Si NWs (3 nm wide, 3 nm high, and 10 nm long) with 1-nm-thick oxide are implanted with As (0.5 keV, 1 × 10^15^ cm^−2^) and annealed at 1,000°C with a hold time of 0 s. Two hundred different random seeds were calculated.

Figure 3 shows random discrete active As distribution in the Si NW calculated by the KMC simulation. The histogram shows the normal distribution curve, and therefore, 200 seeds are large enough to represent the randomness. The average number of active As atoms in the NW is 27 with the standard deviation of 5. Out of 300 As atoms implanted into the 3-nm-wide Si region, only approximately 10% of As atoms are active in the Si NW. Most of the As atoms are in the oxide (approximately 40 atoms), at the oxide/Si interface (approximately 50), in As-vacancy (As-V) clusters (approximately 90), and As precipitates (approximately 90) (see Figure 1). As-V clusters and As precipitates are inactive and immobile. They are formed when As concentration exceeds approximately 10^20^ cm^−3^ (for As-V clusters) and the solubility limit (for As precipitates) [14,15]. In Sentaurus, not only As-V clusters but also As-Si interstitial (I) clusters (inactive and immobile) are taken into account, but As precipitates are not. In the present study, therefore, As-Si interstitial clusters in Sentaurus are interpreted as As precipitates. The calculation results show that the As activation ratio decreases with higher As dose because inactive As species (As-V clusters and As precipitates) are more likely to be formed. In NWs with smaller widths and heights, the As activation is found to be lower because more As atoms are closer to the oxide/Si interface and hence are piled up at the interface.

### NEGF simulation

**Figure 4:**
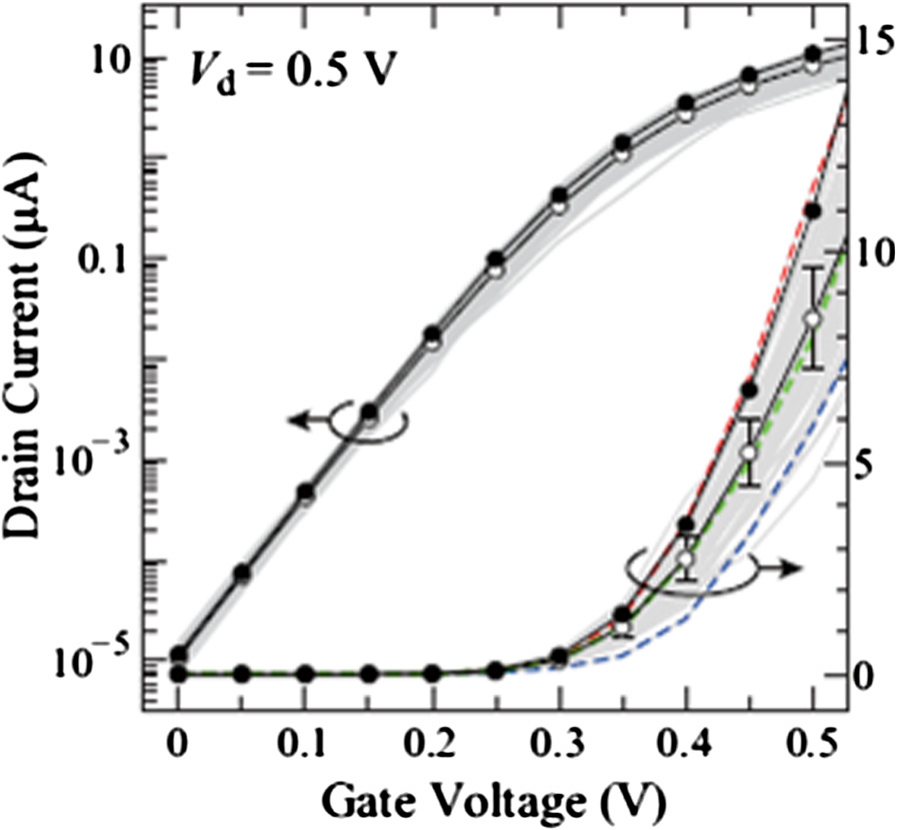
***I***_**d**_**-*V***_**g**_**characteristics of GAA Si NW transistors at*V***_**d**_ **= 0.5 V.** Gray lines show the *I*_d_-*V*_g_ of 100 samples with different discrete As distributions. Open circles represent their average value 〈*I*_d_〉. The continuously doping case with *N*_d_ = 3 × 10^20^ cm^−3^ in the S/D extensions is shown by solid circles for comparison.

**Figure 5:**
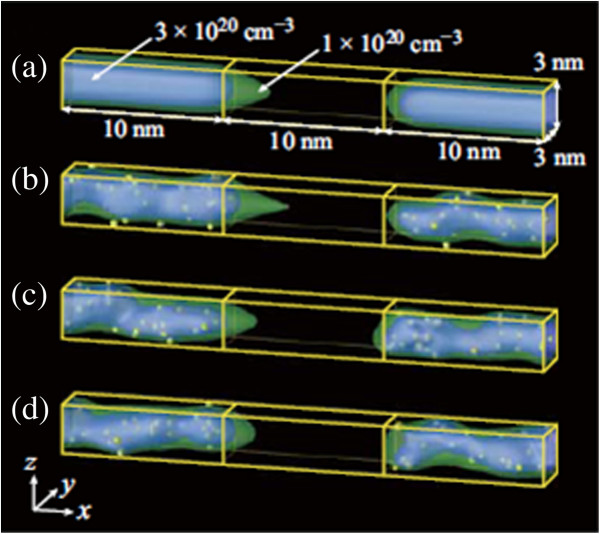
**Carrier density profiles and location of active As atoms in NW devices.** Equidensity surfaces (blue and green surfaces) and dopant positions (yellow dots) for (**a**) continuously doped, (**b**) high-current (red dashed line in Figure 4), (**c**) medium-current (green dashed line in Figure 4), and (**d**) low-current (blue dashed line in Figure 4) devices. *V*_d_ = *V*_g_ = 0.5 V.

**Figure 6:**
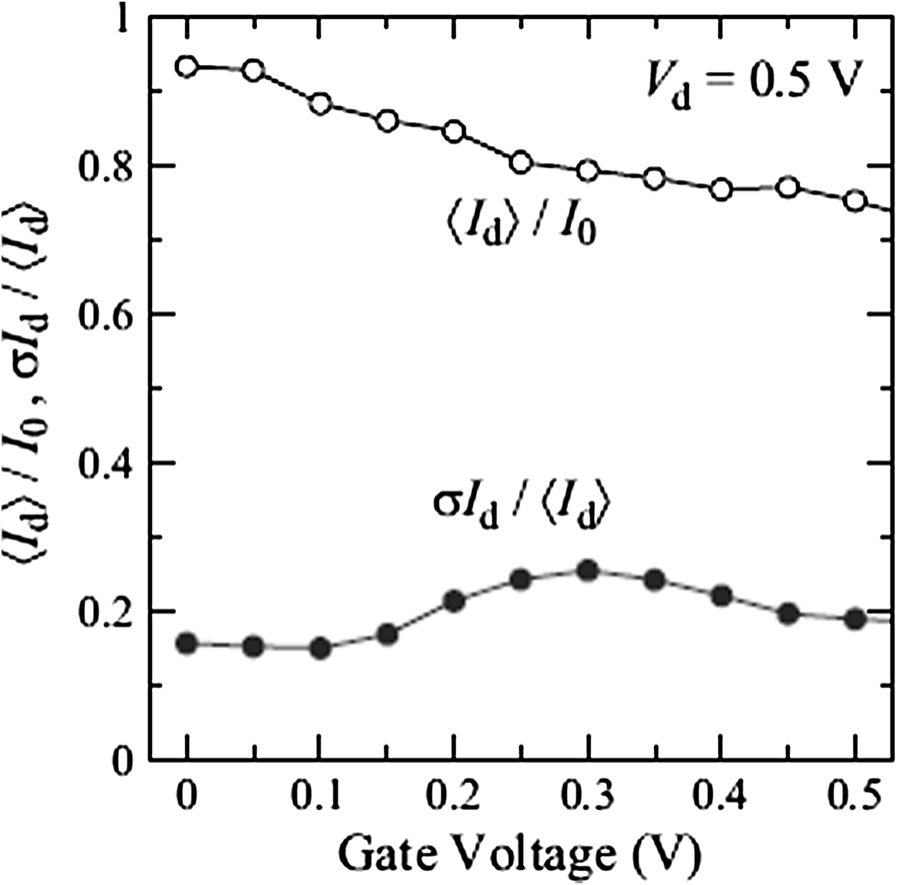
**Average and standard deviation of drain current in NW devices.** Average current 〈*I*_d_〉 and standard deviation *σI*_d_ vs. *V*_g_. *I*_0_ is the drain current of the continuously doped device.

Figure 4 shows the *I*_d_-*V*_g_ characteristics at *V*_d_ = 0.5 V of 100 devices with different discrete As distributions (gray lines). In the figure, their average value 〈*I*_d_〉 (open circles) and the *I*_d_ of a continuously doping case in the S/D extensions (solid circles) are also shown for comparison. For the continuously doping case, the S/D extensions are uniformly n-doped with a concentration of 3 × 10^20^ cm^−3^, which corresponds to the average active As concentration in the Si NWs (see Figure 3). The *I*-*V* characteristics of devices uniformly n-doped with 2 × 10^20^, 2.5× 10^20^, and 3.5 × 10^20^ cm^−3^ are also calculated, and the results show only slight differences (within 10%) compared with the 3 × 10^20^ cm^−3^ case. Figure 5 represents the carrier density profiles and the location of active As atoms in some representative devices. Equidensity surfaces at *V*_d_ = *V*_g_ = 0.5 V (blue and green surfaces for 3 × 10^20^ and 1 × 10^20^ cm^−3^, respectively) and dopant positions (yellow dots) are shown. Figure 5 (a), (b), (c), and (d) correspond to the *I*-*V* characteristics of continuously doped (solid circles in Figure 4), high-current (red dashed line), medium-current (green dashed line), and low-current (blue dashed line) devices, respectively. The drain current of NW devices with random discrete As distribution is found to be reduced compared to that with uniform As distribution. This reduction is ascribed to ionized impurity scattering, which is taken into account for random As distribution, but not for uniform As distribution. The normalized average current 〈*I*_d_〉/*I*_0_ (*I*_0_ is the drain current of the continuously doped device) is found to be approximately 0.8 and decreases with *V*_g_, as shown in Figure 6. The standard deviation of the 100 samples is found to be *σI*_d_ ~ 0.2〈*I*_d_〉.

### Drain current fluctuation

**Figure 7:**
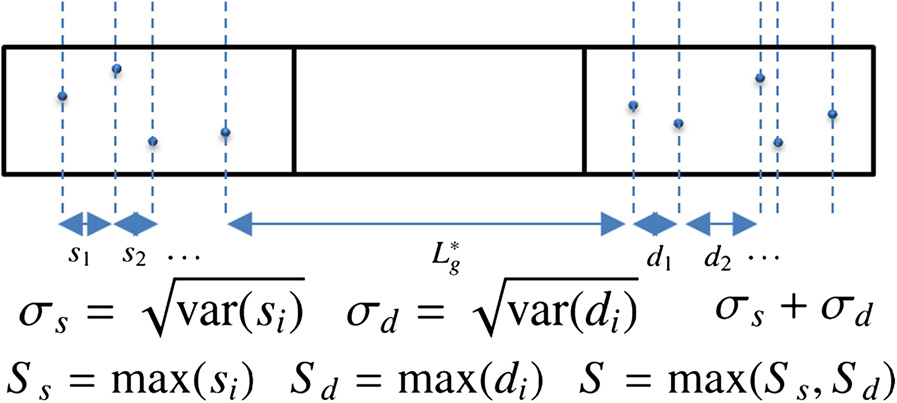
**One-dimensional model to analyze drain current fluctuation.** Blue dots represent active As atoms. *L*_g_^*^, effective gate length; *σ* = *σ*_s_ + *σ*_d_, sum of the standard deviations of interatomic distances in the S/D extensions; *S*_s_, the maximum separation between neighboring impurities in the S extension; *S*_d_, that in the D extension; *S*, that in the S/D extensions. *s*_i_ and *d*_i_ are interatomic distances in the S/D extensions. The effects of the number of As dopants in the S extension (*N*_s_), in the D extension (*N*_d_), and in the S/D extensions (*N*) are also examined.

**Figure 8:**
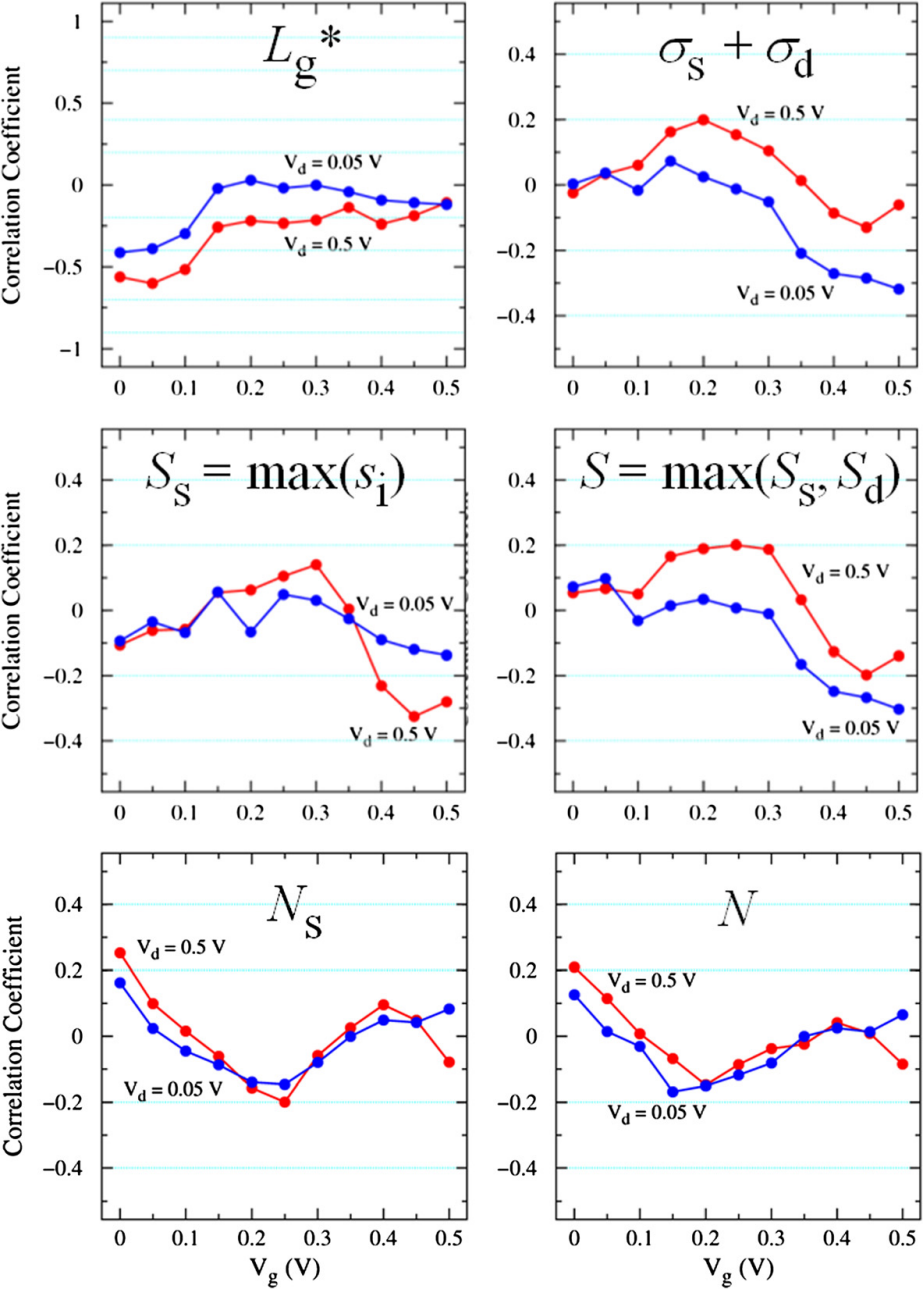
**Correlation coefficients between drain current and factors related to random As distributions.** Blue and red circles represent correlation coefficients at *V*_d_ = 0.05 and 0.5 V, respectively. The coefficient of 0 means no correlation, and those of ±1, the strongest correlation.

In order to investigate the cause of the drain current fluctuation, we examine the correlation between *I*_d_ and the factors related to random As distributions. The factors are extracted from the random As positions, based on a simple one-dimensional model as schematically shown in Figure 7, where blue dots represent active As atoms. The factors are an effective gate length (*L*_g_^*^), standard deviations of interatomic distances in the S/D extensions (*σ*_s_ and *σ*_d_), their sum (*σ* = *σ*_s_ + *σ*_d_), and the maximum separation between neighboring impurities in the S extension (*S*_s_), in the D extension (*S*_d_), and in the S/D extensions (*S*). The effects of the number of As dopants in the S/D extensions are also examined, with the factors of the number of active As in the S extension (*N*_s_), in the D extension (*N*_d_), and in the S/D extensions (*N*). Figure 8 represents the correlation between *I*_d_ and these factors, and Table 1 summarizes the correlation coefficients for the off-state (*V*_g_ = 0 V) and the on-state (*V*_g_ = 0.5 V) at *V*_d_ = 0.05 and 0.5 V. The correlation coefficient *r* is classified as follows: 0.0 < |*r*| < 0.2, little correlation; 0.2 < |*r*| < 0.4, weak correlation; 0.4 < |*r*| < 0.7, significant correlation; 0.7 < |*r*| < 0.9, strong correlation; and 0.9 < |*r*| < 1.0, extremely strong correlation. We highlight clear correlations in Table 1. Note that the threshold voltage is closely related to the off-current because *I*_d_ varies exponentially with *V*_g_ at the subthreshold region.

**Table 1:** Summary of correlation factors of drain current

**Factors**	***V***_**g**_ **= 0.0 V (off-state)**		***V***_**g**_ **= 0.5 V (on-state)**	
	***V***_**d**_ **= 0.05 V**	***V***_**d**_ **= 0.5 V**	***V***_**d**_ **= 0.05 V**	***V***_**d**_ **= 0.5 V**
*L*_g_^*^	*−0.41*	*−0.56*	−0.12	−0.11
*σ*	0.00	−0.02	*−0.32*	−0.06
*S*_s_	−0.09	−0.11	−0.14	*−0.28*
*S*	0.07	0.05	*−0.30*	−0.14
*N*_s_	0.16	0.25	0.08	−0.08
*N*	0.13	0.21	0.07	−0.09

Clear correlations are shown in italics.

Significant correlations between *I*_d_ and *L*_g_^*^ are found at the off-state with *V*_d_ of both 0.05 and 0.5 V. Negative correlation means that *I*_d_ tends to decrease with increasing *L*_g_^*^. The sum of the standard deviations of interatomic distances in the S/D extensions (*σ*) shows a clear correlation at the on-state with *V*_d_ = 0.05 V. Concerning the maximum separation, a clear correlation at the on-state with *V*_d_ = 0.5 V and that with *V*_d_ = 0.05 V are found with *S*_s_ and *S*, respectively, while little correlation with *S*_d_ is seen at any cases. These results demonstrate that the effective gate length (*L*_g_^*^) is a main factor for the off-state, where the channel potential mainly governs the *IV* characteristics. We mention that the off-current becomes larger when active As atoms penetrate into the channel region, which is not taken into account in the present simulation. This increase in off-current can be explained in terms of the ion-induced barrier lowering [16], where the potential barrier in the channel is significantly lowered by attractive donor ions, which enhances the electron injection from the source. For the on-state, random As distribution in the S extension (*S*_s_) is an important factor at high *V*_d_ due to current injection from S, and that in the S/D extensions (*σ* and *S*) is dominant at low *V*_d_ because the back-flow current from D also contributes the current.

On the other hand, little or weak correlations between *I*_d_ and the number of As dopants are found. The weak positive correlations with *N*_s_ and *N* at the off-state are attributed to a tendency that a larger number of dopants lead to smaller *L*_g_^*^. In order to further investigate the effect of the number of As, *I*_d_-*V*_g_ characteristics of NWs implanted at a smaller dose of 2 × 10^14^ cm^−2^ were calculated. The average number of active As atoms in this NW is 16, which averages 1.8 × 10^20^ cm^−3^. The average and standard deviation of the on-current in this NW are almost the same as those in the 1 × 10^15^ cm^−2^ NW. This is consistent with little or weak correlations between *I*_d_ and the number of As dopants as we mentioned above. However, a few out of 100 NW devices of 2 × 10^14^ cm^−2^ have on-current which is only about one half its average. This is attributable to the large interatomic distances of discrete As atoms in these devices. These results indicate that the on-current fluctuation is caused by the fluctuation of interatomic distances of discrete As atoms, not by the fluctuation of the number of As. The off-current fluctuation can be reduced by a process in which dopants in the S/D extensions are likely to exist near the channel region. In contrast, the on-current fluctuation may be inherent in ultra-small NW transistors because interatomic distance is determined by random atomic movement.

## Conclusions

We have theoretically investigated the effects of random discrete distribution of implanted and annealed As atoms in the S/D extensions on the device characteristics of n-type GAA Si NW transistors. KMC simulation is used for generating realistic random distribution of active As atoms in Si NWs, and the current–voltage characteristics are calculated using the NEGF method. The fluctuation of drain current is observed with the normalized standard deviation of approximately 0.2. The correlation between the drain current and the factors related to random As distribution is examined. The results indicate that the on-current fluctuation is not directly due to the fluctuation of the number of dopants in the S/D extensions. The on-current fluctuation may be caused by the randomness of As dopant positions in the S/D extensions and hence is inherent in ultra-small NW transistors.

## Abbreviations

GAA: gate-all-around; KMC: kinetic Monte Carlo; MOSFET: metal-oxide semiconductor field-effect transistors; NEFG: non-equilibrium Green's function; NW: nanowire; RDD: random discrete dopant; S/D: source and drain.

## Competing interests

The authors declare that they have no competing interests.

## Authors’ contributions

MU carried out the KMC calculations to obtain random discrete As distributions in the S/D extensions of NW transistors and drafted the manuscript. KMI supervised the KMC simulation. GM and HM participated in the NEGF simulation of NW transistors. NM carried out the NEGF calculations and analyzed the *I*-*V* characteristics of NW transistors. All authors read and approved the final manuscript.
